# Platelet function suggests cardioembolic aetiology in cryptogenic stroke

**DOI:** 10.1038/s41598-023-32143-0

**Published:** 2023-05-10

**Authors:** Priya Dev, Mohammad Ekhlak, Debabrata Dash, Abhishek Pathak

**Affiliations:** 1 Department of Neurology, Institute of Medical Sciences, Banaras Hindu University, Varanasi, Uttar Pradesh 221005 India; 2 Department of Biochemistry, Center for Advanced Research on Platelet Signaling and Thrombosis Biology, Institute of Medical Sciences, Banaras Hindu University, Varanasi, Uttar Pradesh India

**Keywords:** Stroke, Stroke

## Abstract

Platelet-monocyte (PMA) and platelet-neutrophil aggregations (PNA) play critical roles in the evolution of acute ischemic stroke (AIS). The present study investigates the mechanistic basis of platelet responsiveness in cryptogenic stroke compared with cardioembolic stroke. Platelet from 16 subjects, each from cryptogenic and cardioembolic stroke groups and 18 age-matched healthy controls were subjected to different investigations. Compared to healthy controls, platelet-monocyte and platelet-neutrophil interactions were significantly elevated in cryptogenic (2.7 and 2.1 times) and cardioembolic stroke (3.9 and 2.4 times). P-selectin expression on platelet surface was 1.89 and 2.59 times higher in cryptogenic and cardioembolic strokes, respectively, compared to healthy control. Cell population with [Ca^2+^_i_] in either stroke group was significantly outnumbered (by 83% and 72%, respectively, in cryptogenic and cardioembolic stroke) in comparison to healthy controls. Noteworthy, TEG experiment revealed that the cryptogenic stroke exhibited significant decline in Reaction Time (R) and amplitude of 20 mm (K) (by 32% and 33%, respectively) while thrombin burst (α-angle) was augmented by 12%, which reflected substantial boost in thrombus formation in cryptogenic stroke. Although TEG analysis reveals a state of hypercoagulability in patients with cryptogenic stroke. However, platelets from both stroke subtypes switch to a ‘hyperactive’ phenotype.

## Introduction

A highly debilitating illness, stroke is currently one of the most common causes of health impairment worldwide^1^. In 2019, the global prevalence of stroke was 101.5 million people that included 77.2 million diagnosed with ischemic stroke, and 3.3 million deaths from ischemic pathology^2^. The etiopathogenesis of stroke is linked to myriad of factors, including age, gender, chronic illnesses such as diabetes and hypertension, cardiovascular diseases, smoking and lifestyle variables like obesity and stress^3–5^. According to Trial of ORG10172 in Acute Stroke Treatment (TOAST) classification, ischemic stroke has been subdivided into various subtypes such as large artery atherosclerosis (LAA), small vessel occlusion (SVO), cardioembolic (CE) stroke, and stroke of undetermined aetiology (cryptogenic stroke). LAA is diagnosed if more than 50% of large arteries supplying brain is stenosed while SVO is associated with the lacunar infarct size less than 1.5 cm. Cardioembolic stroke develops due to an embolus arising from the heart whereas cryptogenic stroke does not have a known aetiology^6^. Blood stasis rather than platelet hyperfunction has been implicated in pathogenesis of cardioembolic stroke^7^, which is associated with raised concentration of fibrinogen, d-dimer, and von Willebrand factor in blood serum indicative of prothrombotic state^8^. Notably, cryptogenic stroke accounts for approximately 15–40% of all cases of ischemic stroke^9,10^. The exact pathophysiology of this subtype remains obscure though underlying cardioembolic origin and hypercoagulability have been speculated.

Several studies have associated elevated serum levels of brain natriuretic peptide (BNP) and N-terminal pro BNP (NT-pro BNP) in cryptogenic stroke^9^. In a recent meta-analysis of 2834 patients by Llombart et al., serum levels of BNP and NT-proBNP were found to be significantly raised in patients with cardioembolic stroke^10,11^. During development of atherosclerosis, activated platelets interact with endothelial cells leading to arterial thrombogenesis and leucocyte activation^12,13^. Formation of platelet-leukocyte aggregates (PLA) mediated through P-selectin-PSGL-1 interaction represents an essential mechanism by which leukocytes contribute to thrombotic and inflammatory events. PLA is further stabilised by crosstalk between numerous additional ligands with their cognate receptors that triggers release of granule content by both the interacting cells, thus modulating leukocyte function and fine-tuning immune responses^14^.

P-selectin is a transmembrane glycoprotein stored in the Weibel-Palade bodies of vascular endothelial cells and in α-granules of platelets.^15,16^. Expression of P-selectin on surface membrane is significantly enhanced in activated platelets^17^. Previous research has demonstrated that activation markers like P-selectin are upregulated at various stages following ischemic stroke and transient ischemic attack^18–21^, associated with rise in blood–brain barrier permeability and increase in infarct volume, thus affecting the severity of stroke^22–24^. Platelet surface glycoproteins (GP) cluster of differentiation, CD-41/CD61 (integrins α_IIb_β_3_) play crucial role in mediating platelet aggregation^25^. CD14 is a myeloid cell differentiation antigen expressed on surfaces of monocytes and neutrophils and attached to the membrane through a glycosylphosphatidylinositol (GPI) anchor^26^. Many thrombo-inflammatory diseases, including ischemic stroke, have elevated PLA.

The molecular underpinnings of platelet activation in ischemic stroke remain elusive. A transient rise in cytosolic free Ca^2+^, [Ca^2+^]_i_, is presumed to be critical in triggering temporary platelet adhesion whereas sustained calcium signal is necessary for irreversible platelet aggregation and thrombosis^27^. Apart from platelet hyperactivity, hypercoagulability is, too, associated with a heightened risk of cerebrovascular accidents, including ischemic stroke^28^. Several studies have attributed greater platelet reactivity, higher blood level of fibrinogen, and/or stimulated coagulation system to higher incidence of recurrent vascular events^18,29^.

Notably, most studies on platelet hyperactivity in stroke focus on ischemic stroke though there is dearth of serious research on the cryptogenic stroke subtype. Lack of information on underlying molecular details leading to platelet hyper-responsiveness is the major impediment in developing effective anti-thrombotic and anti-platelet therapeutic measures. Since platelet hyperactivity could either be the cause or consequence of thrombosis in cryptogenic stroke, we chose cardioembolic stroke as the control group where aetiology is related to blood stasis rather than platelet hyperactivity while being associated with thrombosis. In the present study, we have investigated platelet responsiveness in cryptogenic stroke compared to cardioembolic stroke and have evaluated agonist-induced platelet responses in both groups.

## Material and methods

### Materials

Thrombin receptor activating peptide, TRAP (# S1820) was the product of Sigma. Anti-CD62P (# 550561), anti-CD14 (# 555397) and anti-CD41a (#559777) antibodies, and BD FACS Lysing Solution (# 349202) were from BD Biosciences. Kaolin vials along with cups and pins (# 39235010) were purchased from Haemonetics. Fluo-4/AM (# F14201) was the product of Invitrogen. All other reagents were of analytical grade. Type I deionized water (18.2 MΩ.cm, Millipore) was used for preparation of solutions.

### Patient selection

All the patients were evaluated keeping with the latest American Heart Association/American Stroke Association guideline for stroke management. A complete evaluation, including clinical assessment and neurological examination, was carried out in line with the National Institutes of Health Stroke Scale (NIHSS) within 60 min of the patient’s arrival. The patient underwent a diagnostic evaluation to establish stroke type and etiological classification as per TOAST. Complete clinical and demographic profile, as well as vascular risk factors (blood pressure > 140/90 mm Hg at two readings before stroke or 15 days after stroke or on an antihypertensive regimen, elevated haemoglobin A1c or elevated blood glucose at two lessons before or 15 days after stroke or on an anti-diabetic treatment, total cholesterol 150–250 mg/dl or triglycerides 10–250 mg/dl or a lipid-lowering medication) were recorded.

The diagnostic evaluation included non-contrast computed tomography (NCCT) or brain magnetic resonance imaging (MRI), contrast MR Angiography (vascular imaging (of intra- and extracranial vessels)), hemogram, biochemical tests, electrocardiogram, transoesophageal echocardiography, and assessment of prothrombotic state. The patient also underwent 24 h Holter monitoring to rule out any atrial fibrillation. ‘Cryptogenic stroke’ refers to stroke where no definite cause was identified. In contrast, cardioembolic stroke was defined as stroke with underlying cardiac pathology such as atrial fibrillation, rheumatic heart disease etc. The details have been added in Supplementary Fig. 1. None of the patients was on prior antithrombotics or statins. In the cardioembolic population, 7 patients were rheumatic heart disease with mitral valve stenosis, 7 were Non-valvular Atrial fibrillation, and 2 were of ischemic dilated cardiomyopathy. The details of the clinical and biochemical profiles of the selected patients can be seen in Supplementary Table 1.

### Sample preparation

Blood from patients diagnosed as cryptogenic stroke (n = 16) and cardioembolic stroke (n = 16) was collected within seven days of stroke onset from antecubital venepuncture with minimal stasis to avoid platelet stimulation. Our patients at the time of recruitment were not on statins, but after the samples were drawn, patients were started on statins. Sample from age-matched healthy controls (n = 18) with no relevant disease or medication history was collected within a year. The study methodologies conformed to the standards set by the Declaration of Helsinki. Blood was processed within 20 min of collection^28^. Whole blood was employed for platelet-leucocyte interaction and thromboelastography experiments. Blood was centrifuged at 100×*g* for 20 min at room temperature to obtain platelet-rich plasma (PRP), used for P-selectin externalisation and intracellular calcium measurement studies.

### Study of platelet-monocyte and platelet-neutrophil aggregation

Fresh human blood (20 µl) was added to a cocktail containing 10 µl each from APC-anti-CD41a (platelet-specific) and FITC-anti-CD14 (leukocyte-specific) antibodies and mixed gently. Samples were incubated with TRAP (5 µM) for 15 min at RT. RBCs were lysed with 800 µl FACS lysis solution (1×, BD Biosciences) for 10 min at RT^30,31^.

Leucocyte-platelet interaction was analysed on a flow cytometer (FACS Calibur, BD Biosciences) within 30 min. Side scatter (SSC) voltage was set at 350 with a threshold of 52 V, and amorphous gates were drawn to encompass neutrophils and monocytes separate from noise. A dot plot of SSC versus log FITC-CD14 fluorescence was created in the CellQuest Pro software. Amorphous gates were drawn for monocyte (high fluorescence and low SSC) and neutrophil (low fluorescence and high SSC) populations. All fluorescence data from each sample were collected using 4-quadrant logarithmic amplification for 1000 events in either neutrophil or monocyte gate and analysed using CellQuest Pro Software.

### Secretion from platelet α-granules

Secretion from platelet α-granules was quantified in circulating platelets from healthy control as well as cryptogenic and cardioembolic stroke by measurement of surface expression of P-selectin (CD62P)^32,33^. Briefly, platelets (2.5 × 10^8^/ml) in PRP were stained with PE-labelled anti-CD62P antibody (5% v/v) for 30 min at RT in the dark. Samples were suspended in sheath fluid and subjected to flow cytometry. An amorphous gate was drawn to separate noise from the platelets population. Fluorescence data were derived from quadrant logarithmic amplification for 10,000 events in platelet gate from each sample and analysed using CellQuest Pro software.

### Measurement of intracellular free calcium

Platelet count was maintained at 2 × 10^6^/ml by diluting PRP (5 µl) with 495 µl of 1X Tyrode’s buffer (HEPES 20 mM, NaCl 134 mM, KCl 2.9 mM, MgCl_2_ 1 mM, NaH_2_PO_4_ 0.34 mM, NaHCO_3_ 12 mM) supplemented with 5 mM glucose, followed by incubation with Fluo-4/AM (5 µM) for 30 min at RT in dark. After appropriate gating of platelets using a flow cytometer (Becton Dickinson, model Accuri C6), events were analysed in the FL1 channel. Fluo-4 baseline fluorescence was recorded for 1.5 min, followed by the addition of TRAP (5 µM) using gel-loading tips as described earlier^18^.

### Thromboelastography (TEG)

Coagulation parameters in whole blood were studied by employing Thromboelastograph 5000 Hemostasis Analyzer System (Haemonetics) and TEG analytical software. Fresh blood (1 ml) was taken in citrated kaolin vials and mixed gently. CaCl_2_ (20 µl) was added to 340 µl sample to initiate coagulation cascade^34^. The mixture was placed in disposable TEG cups. Data were collected as per manufacturer’s instructions until maximum amplitude was reached or 60 min had elapsed. Results presented graphically reveals the following: Reaction Time (R) in min, time until the first clot is detected; Clot Kinetics (K) in min, time taken to achieve a level of clot strength (amplitude of 20 mm), i.e. amplification; α-angle, rate of clot formation or ‘thrombin burst’; Maximum Amplitude (MA), maximum strength and firmness of the clot; and lysis at 30 min (LY30), percentage of lysed clot after 30 min.

### Statistical evaluation

Data are presented as either means ± S.D. or means ± SEM of at least five independent experiments from different blood donors. Statistical analyses were performed using GraphPad Prism, version 8.4.0. Ordinary one-way analysis of variance (ANOVA) with Sidak’s multiple comparison tests were used for statistical evaluation. Tests were considered significant at *p* < 0.05.

### Ethics approval and consent to participate

Blood samples were drawn from human participants under written informed consent strictly as per recommendations and approval of the Institutional Ethical Committee of the Institute of Medical Sciences, Banaras Hindu University. The study methodologies conformed to the standards set by the Declaration of Helsinki.

## Results

The demographic data of the patients have been represented in Supplementary Table 1.

### Increased platelet-leukocyte aggregates in circulation from patients with cryptogenic and cardioembolic stroke

**Figure 1:**
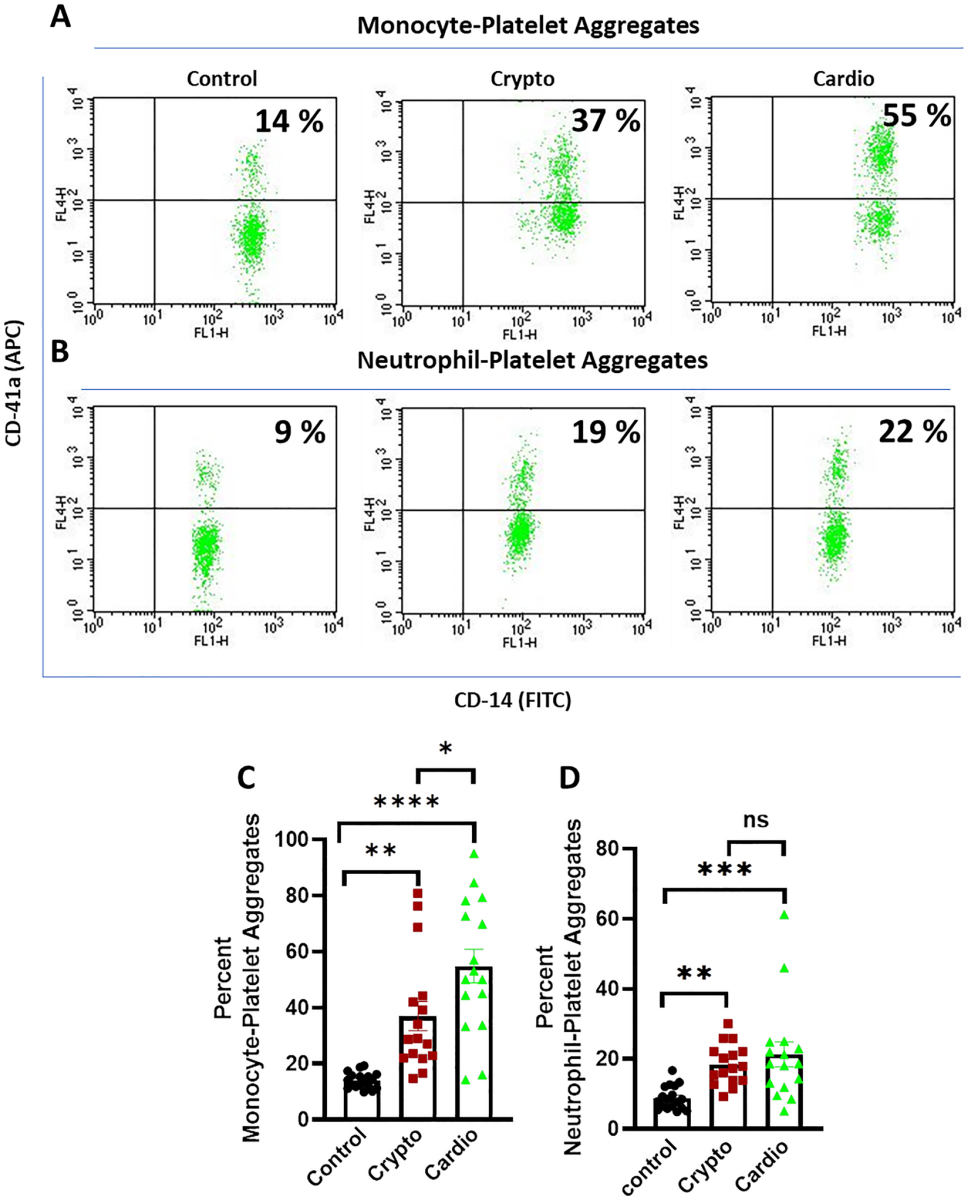
Detection of platelet-monocyte and platelet-neutrophil aggregates in circulation: (**A,B**) flow cytometric analysis of monocyte-platelet and neutrophil-platelet aggregates, respectively, in whole blood stained with platelet-specific marker, anti-CD41a-APC and neutrophil/monocyte-specific marker, anti-CD14-FITC. Samples were obtained from control and different stroke groups as indicated. Amorphous gates were drawn for monocyte (high fluorescence and low SSC) and neutrophil (low fluorescence and high SSC) populations. Numbers in right upper quadrant represent percent of aggregates. (**C,D**) corresponding scatter plot bar diagrams representing mean percent of PMA and PNA, respectively Data are represented as mean ± SEM of at least 16 different experiments. *p < 0.05, **p < 0.01, ***p < 0.001 and ****p < 0.0001. *ns* non-significant.

Presence of platelet-monocyte and platelet-neutrophil aggregates in circulation of acute phase patients was analyzed within seven days of onset of stroke. Significantly higher level of circulating aggregates was observed in stroke patients compared to healthy controls (Fig. 1). The mean values for PMA were found to be 13.94, 36.98 and 54.8 for controls, cryptogenic and cardioembolic stroke groups whereas those for PNA were 8.78, 18.43, 21.29, respectively. Circulating PMA was 2.7 and 3.9 times higher in cryptogenic and cardioembolic stroke groups, respectively, compared to healthy controls. PNA was also 2.1 times higher in cryptogenic stroke and 2.4 times higher in cardioembolic stroke compared to controls. Remarkably, percentages of circulating PMA were found to be significantly higher in cardioembolic stroke in comparison to cryptogenic stroke. However, this was not significant in case of PNA (Fig. 1C,D).

### Increased platelet activation in patients with cryptogenic and cardioembolic stroke

**Figure 2:**
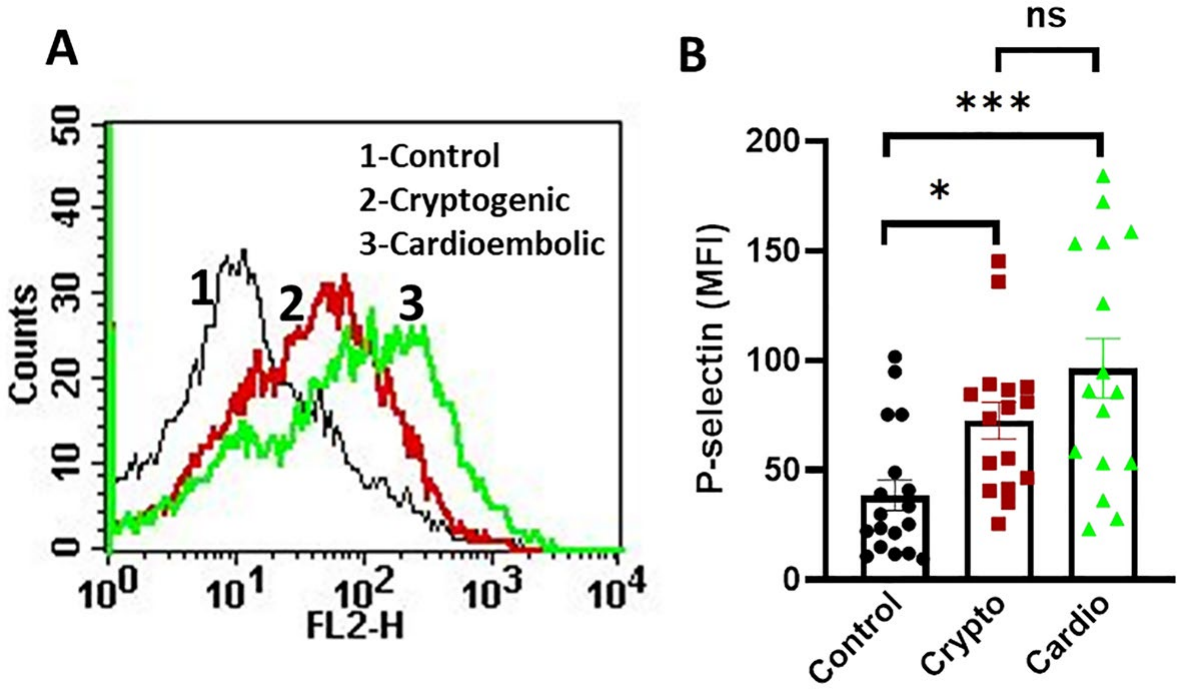
Exposure of P-selectin on surface of unstimulated platelets: (**A**) flow cytometric analysis of binding of PE-anti-P-selectin antibody to surface of unstimulated platelets from different stroke groups as indicated. (**B**) Corresponding scatter plot bar diagram representing mean fluorescence intensity. Data are presented as mean ± SEM of at least 16 different experiments. *p < 0.05 and *** p < 0.001. *ns* non-significant.

We evaluated the surface expression of the P-selectin (CD62P), a potent marker for platelet activation and secretion from α granules, in platelets obtained from either stroke groups. The cryptogenic and cardioembolic stroke patients presented with augmented surface expression of P-selectin in platelets compared with control counterparts (Fig. 2), suggestive of ‘hyperactive’ platelets in circulation in stroke groups. The mean values for P-selectin expression in control (n = 18), cryptogenic (n = 16) and cardioembolic stroke (n = 16) groups were found to be 38.48, 72.60, and 96.58 mean fluorescence intensity (MFI), respectively (Fig. 2B). However, platelet surface exposure of P-selectin was not found to be significantly different between cryptogenic and cardioembolic stroke patients.

### Raised intracellular Ca^2+ ^in circulating platelets from patients with cryptogenic and cardioembolic stroke

**Figure 3:**
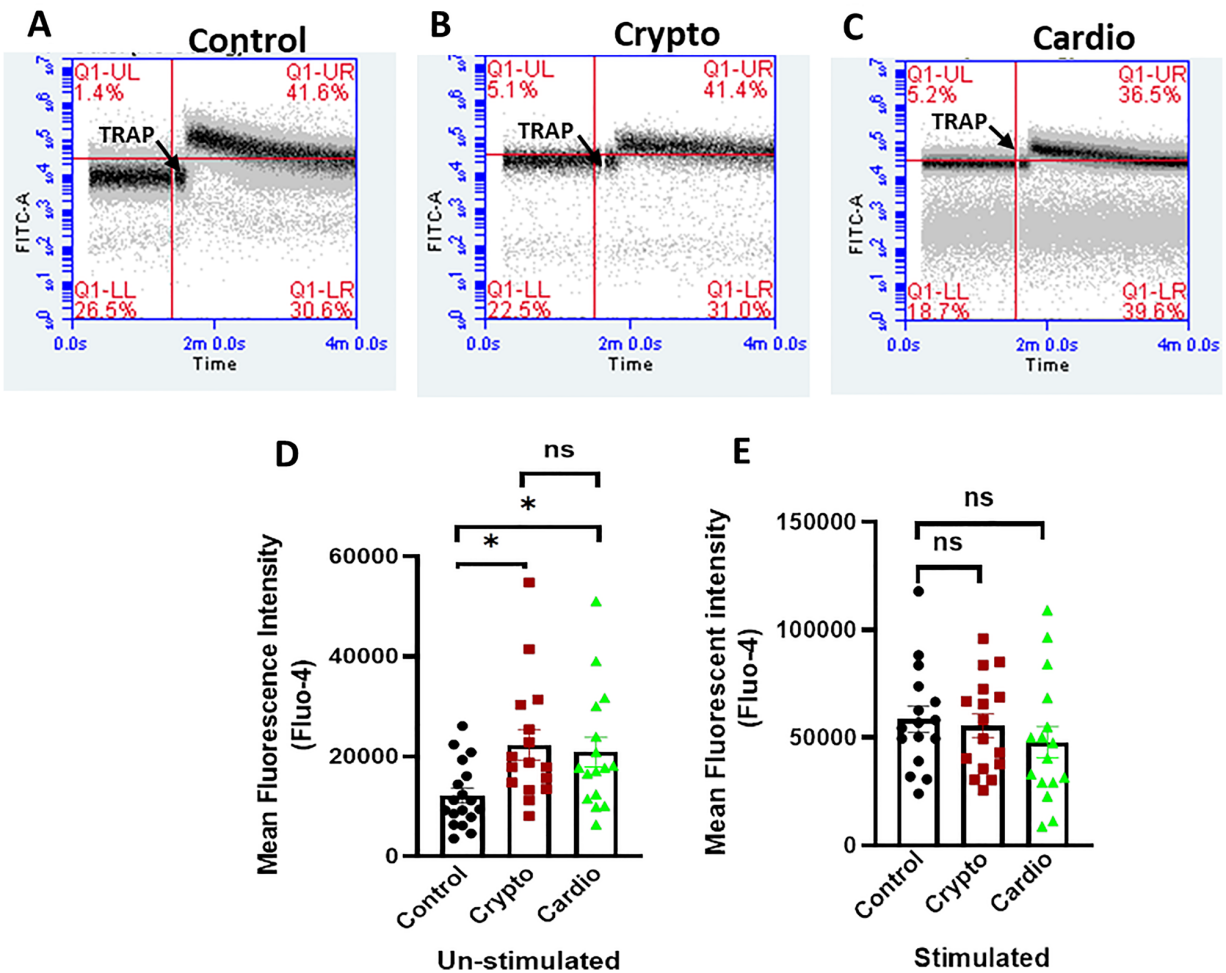
Determination of platelet population with raised intracellular calcium: (**A–C**) Flow cytometry of Fluo-4-labeled platelets representing cell population with high fluorescence intensity in control (n = 18), cryptogenic (n = 16) and cardioembolic stroke (n = 16) groups, respectively. (**D**) Scatter plot bar diagram representing mean fluorescence intensity over 1.5 min of a population before TRAP addition. (**E**) Scatter plot bar diagram representing mean fluorescence intensity over 1.5 min of a population after TRAP addition. Data are presented as mean ± SEM of at least 16 different experiments. *p < 0.05. *ns* non-significant.

As free intracellular calcium is a critical regulator of platelet functions, we next evaluated [Ca^2+^]_i_ in Fluo-4-loaded platelets from cryptogenic stroke (N = 16) and cardioembolic stroke (N = 16) before and after agonist challenge by flow cytometry and compared both the groups with healthy control (n = 18). Fluo-4-loaded platelet suspension was pre-supplemented with 1 mM calcium and fluorescence was recorded for 1.5 min (Fig. 3). The unstimulated platelets from cryptogenic and cardioembolic stroke groups exhibited significantly raised [Ca^2+^]_i_ compared to healthy controls (by 83.37% and 71.60%, respectively), reflective of higher intracellular calcium in circulating platelets from stroke, though there was no significant difference in values between the two stroke groups (Fig. 3). As expected, addition of TRAP (5 µM) to the cell suspension shows remarkable rise in fluorescence of Fluo-4-positive events in healthy control (by 5.37-fold). However, no further boost in cytosolic calcium level was observed in platelets from stroke cases following agonist challenge (Fig. 3E).

### Hypercoagulability in patients with cryptogenic stroke

**Figure 4:**
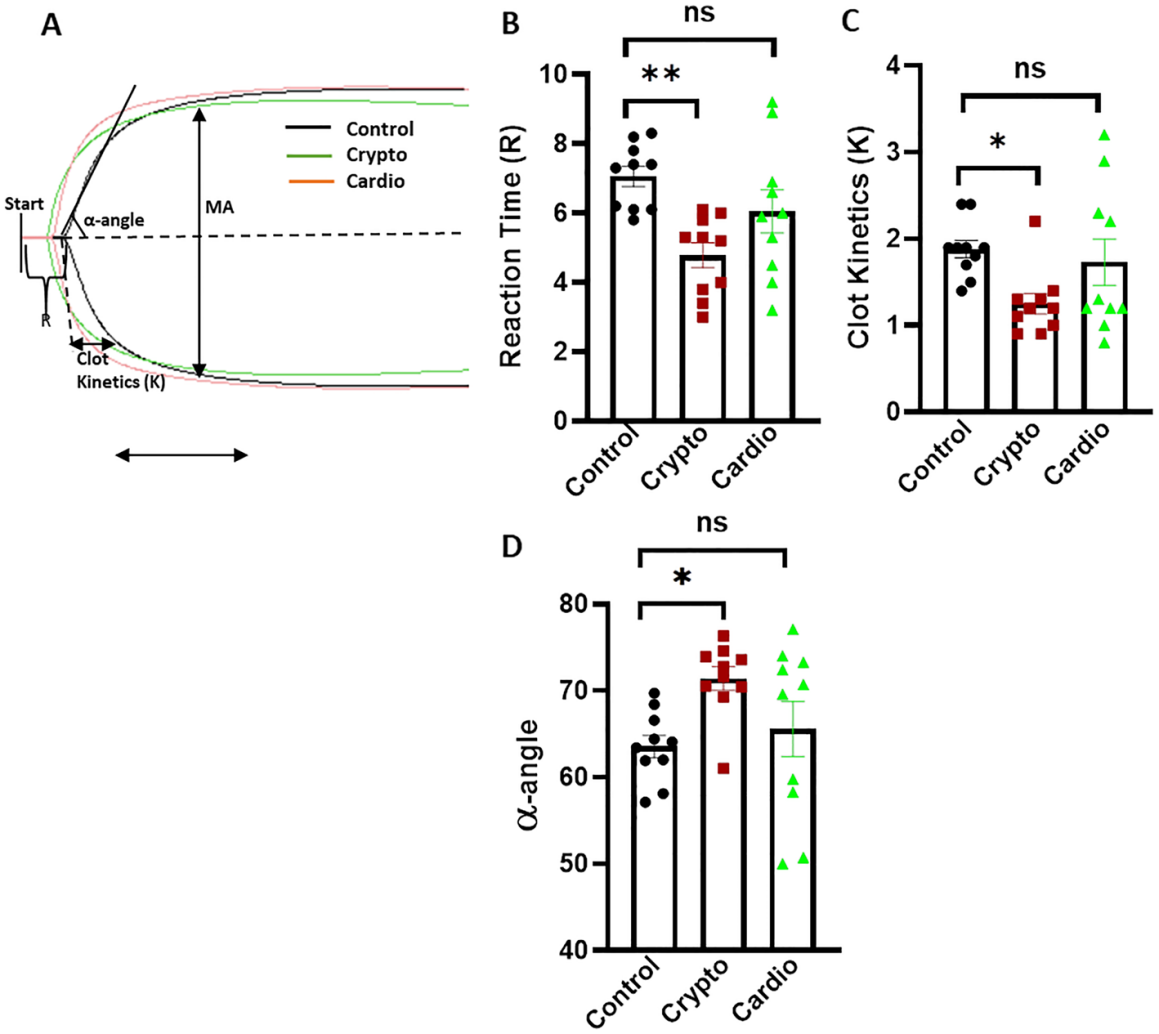
Determination of hypercoagulability and thrombus stability: (**A**) representative thromboelastogram of kaolin-stimulated citrated whole blood obtained from healthy controls, cryptogenic and cardioembolic stroke patients, as indicated. (**B–D**) Corresponding scatter plot bar diagrams representing Reaction Time (R), Clot Kinetics (K) and α-Angle, respectively, from 10 healthy volunteers and 10 patients from each stroke group. Data are represented as mean ± SEM of at least 10 different experiments. *p < 0.05 and **p < 0.01. *ns* non-significant.

Kaolin-activated thromboelastography is a measure of clot strength and the intrinsic pathway of blood coagulation. In order to validate platelet hyper responsiveness, we performed TEG in 10 healthy controls and 10 age-matched counterparts each from cryptogenic and cardioembolic stroke groups (Fig. 4). Sample from cryptogenic stroke exhibited significant drop in Reaction Time (R) (by 32.15%) and Clot Kinetics (K) (by 33.51%), associated with increase in α-Angle (by 12.31%) compared to the healthy controls, which were reflective of hypercoagulability in the cryptogenic stroke group. No difference was observed in Maximum Amplitude (MA) between the two groups. TEG parameters did not differ significantly between the cardioembolic stroke vs control groups though there was a tendency towards augmented clot strengthening and firmness in former.

## Discussion

In this report, we have extensively studied platelet responsiveness in cryptogenic stroke and compared it with the cardioembolic stroke groups of patients to understand the pathogenesis of the former. Platelet-monocyte and platelet-neutrophil aggregates in circulation, platelet intracellular free calcium and surface P-selectin were statistically higher in the patients of cryptogenic and cardioembolic stroke than in healthy controls. However, when compared between both the stroke groups, these parameters were found to be insignificant except PMA, which was statistically higher in the cardioembolic stroke patients. At the same time, analysis of coagulation dynamics (R, K and α-angle) from thromboelastography studies revealed hypercoagulable blood in cryptogenic stroke compared to control. Despite the tendency towards hypercoagulability, the cardioembolic stroke did not register a significant difference from the control.

In healthy humans, neutrophils represent the dominant leukocyte subpopulation with about 40–70%, followed by lymphocytes (20–45%) and monocytes (2–10%). As circulating, platelets from stroke have higher expression of P-selectin on their surface and due to affinity between P-selectin and PSGL-1 on the surface of leukocytes, platelet-leucocyte aggregates circulate significantly larger number in stroke. Circulating level of PLAs significantly increase within 24 h after the onset acute ischemic stroke and represents a more sensitive marker for platelet activation compared to parameters like platelet surface P-selectin and platelet aggregation^35^. Furthermore, in patients with large-artery atherosclerosis-induced stroke, aspirin and clopidogrel reduce platelet aggregation and PLA formation more efficiently than aspirin alone^14^. These results indicate that inhibiting several platelet activation pathways might be beneficial in cryptogenic stroke patients by limiting ischemic stroke recurrence and neurologic deterioration.

Several studies involving whole blood flow cytometric analysis of platelet activation markers such as P-selectin have suggested excessive activation of platelets in the acute phase of ischemic stroke^18,35^. It has been postulated that platelet-monocyte aggregate is a more sensitive marker of platelet activation than P-selectin in myocardial infarction patients^36^. Although platelet activation markers were significantly higher in both the stroke subgroups than the control group, they were found to be insignificant when the two subgroups were compared among themselves. These results are in keeping with the reports of Oberheiden et al. and Li Tao et al. for cardioembolic stroke^37,38^. Similarly, Marquardt et al., demonstrated that both PNA and PMA are significantly higher in acute stroke patients than in control. At the same time, platelet activation markers have previously never been adequately studied in the cryptogenic stroke population. There was no difference between the two stroke subgroups in platelet activation and platelet-leucocyte interaction, which is a significant outcome from the present study^39^.

In contrast to Zeller et al., our result demonstrated that acute cardioembolic stroke did not significantly differ in platelet activation markers (P-selectin) compared to control. This variance could be explained from the difference in the selection of the patients^40^. In another study by Tsai et al., platelet activation markers and PLA in non-cardioembolic stroke were significantly higher than control^6^. In similar studies, Marquardt and colleagues have shown that both PMA and PNA are increased in the acute phase of ischemic stroke compared to control^40^. Our study also points out that there was no significant difference in platelet-leucocyte aggregate formation and surface abundance of P-selectin when cardioembolic stroke was compared with cryptogenic stroke. This partially agrees with the study published by Turgut et al., they showed that platelet activation markers were significantly more conspicuous in non-cardioembolic stroke vis-à-vis cardioembolic stroke^41^. However, there was no difference in P-selectin expression in the cardioembolic vs. non-cardioembolic group, which is consistent with our results^41^. We have also demonstrated that the platelet-monocyte aggregate is significantly higher in cardioembolic stroke, which contradicts that published by Cerlleti et al. This difference could be due to the different patient population; Cerlleti had chosen cases predominantly with atrial fibrillation while our group had a small number of patients with atrial fibrillation^35^. Our findings do reiterate that platelet activation markers have the essential underlying pathogenic role in both cryptogenic and cardioembolic stroke. Our study has also shown that, P-selectin surface abundance was significantly higher in the stroke subgroup compared to the control group to establish its role as a platelet activation marker. This result denotes that the underlying pathogenesis might be the same in both groups. Platelet activation markers in both subgroups of stroke patients were not different. Whether this was the effect or the cause of stroke cannot be established. In a different study by Michael et al., found that in experimental stroke in mice lacking CD84, a homophilic immunoreceptor of the SLAM family, on either platelets or T cells displayed reduced cerebral CD4+ T cell infiltration and thrombotic activity^42^. In addition, a review by Younes Zaid also suggested that platelet play an active role in inflammation and immunity^43^. Thus, inhibiting platelets in both subtypes may have additional benefits.

The thromboelastography results clearly demonstrate that the time till first clot formation and clot kinetics are significantly shorter in the cryptogenic stroke group than in the healthy control, associated with an increase in rate of clot formation (α-Angle), which were strongly suggestive of hypercoagulability. Oral anticoagulation is the drug of choice in cardioembolic stroke, and these results suggest that oral anticoagulants may help prevent stroke recurrence in the cryptogenic population.

Our study has significant strengths. First, this has been the first study looking into the platelet activation markers in cryptogenic stroke and comparing it with the cardioembolic stroke group to look for the difference. Second, we did extensive tests to examine platelet activation markers, including TEG, interaction with leucocytes, P-selectin surface exposure, and intracellular calcium measurement studies. Third, our results might help understand the basic pathophysiology of cardioembolic stroke. Results suggest the need for clinical trials of anticoagulation as a therapeutic role in the cryptogenic subgroup of stroke patients in preventing future stroke. However, a limitation of this study has been the relatively smaller population size. We have excluded the sick patients requiring critical care, limiting the external validity of the study results. The guidelines for mild to moderate ischemic stroke mention using dual antiplatelets for three weeks in cryptogenic stroke, while a patient needs anticoagulation in case of cardioembolic stroke^44^. Therefore each patient of cryptogenic stroke must undergo a proper evaluation of the aetiology of the ischemic stroke.

Our study highlights the importance that the cryptogenic stroke population may not be essentially “cryptogenic” and may have an underlying cardioembolic phenomenon that needs to be proven in future studies in larger population.

## Supplementary Information

Supplementary Information.

## Data Availability

All data generated or analysed during this study are included in this published article (and its Supplementary Information files).
