# Supplementary material for: Platelet function suggests cardioembolic aetiology in cryptogenic stroke
Source: Sci Rep. 2023 May 10;13:7615. doi: 10.1038/s41598-023-32143-0 (PMC10172292; doi:10.1038/s41598-023-32143-0)

Supplementary Table 1: Demographic and clinico-biological data of cryptogenic and cardioembolic

Characteristics	Cryptogenic Stroke (N=16)	Cardioembolic Stroke (N=16)
Age (Mean ±SD)	64.19±12.41	61.63±14.11
Gender (M/F)	14/2	14/2
Ischemic stroke classification (TACI/ PACI/ LACI/ POCI)	0/15/1/0	0/14/1/1
Hypertensive	9	10
Diabetic	4	6
Dyslipidaemia	2	1
NIH Stroke Scale (NIHSS)	14.94±7.9	14.13±5.3
mRS (Degree of disability)	4.63±0.72	4.44±0.73
Blood Investigations RBS	146.96 ± 47.17	127.68 ± 29.99
Total cholestrol	199.78 ± 51.28	133.54 ± 25.29
LDL	108.54 ± 36.24	75.29 ± 20.76
HDL	48.59 ± 15.83	37.09 ± 11.44
VLDL	59.55 ± 53.05	33.33 ± 31.16
TG	121.33 ± 69.70	164.96 ± 156.39
Blood urea	46.76 ± 23.55	38.47 ± 17.08
Serum Creatinine	1.06 ± 0.38	0.83 ± 0.27

Na	137.40 ± 3.47	131.62 ± 27.69
K	4.27 ± 0.75	4.16 ± 1.16
Cl	104.65 ± 4.51	103.41 ± 3.50
SGOT	55.68 ± 59.48	48.86 ± 28.94
SGPT	37.71 ± 21.85	46.41 ± 34.25
Alkaline Phosphatase	154 ± 82.27	194.91 ± 55.28
Serum protein	7.37 ± 0.70	7.12 ± 0.59
serum albumin	4 ± 0.56	3.66 ± 0.38
Total bilirubin	0.93 ± 0.46	0.69 ± 0.28
Direct bilirubin	0.30 ± 0.14	0.30 ± 0.13
Indirect bilirubin	0.62 ± 0.39	0.38 ± 0.19
TLC	11.69 ± 3.80	9.57 ± 2.63
RBC	4.47 ± 0.65	4.49 ± 0.49
HGB	19.40 ± 27.85	12.07 ± 1.48
PLT	188.87 ± 47.55	176.01 ± 74.64

* TACI-Total anterior circulation infarct; PACI-Partial anterior circulation infarct ; LACI-Lacunar Cerebral Infarct; POCI- Posterior circulation infarct; NIHSS- National institute of health stroke scale; mRS-Modified rankin scale; RBS-Random blood sugar; LDL-Low density lipoprotein; HDL-High density lipoprotein; VLDL-; TG-Triglyceride; Na-Sodium; K-Potassium; Cl-Chlorine; SGOT- Serum glutamic-oxaloacetic transaminase; SGPT- Serum glutamic pyruvic transaminase; TLC-Total leukocyte count; RBC-Red blood cells; HGB-Haemoglobin; PLT-Platelet.

Supplementary Figure 1: Etiological classification of ischemic stroke

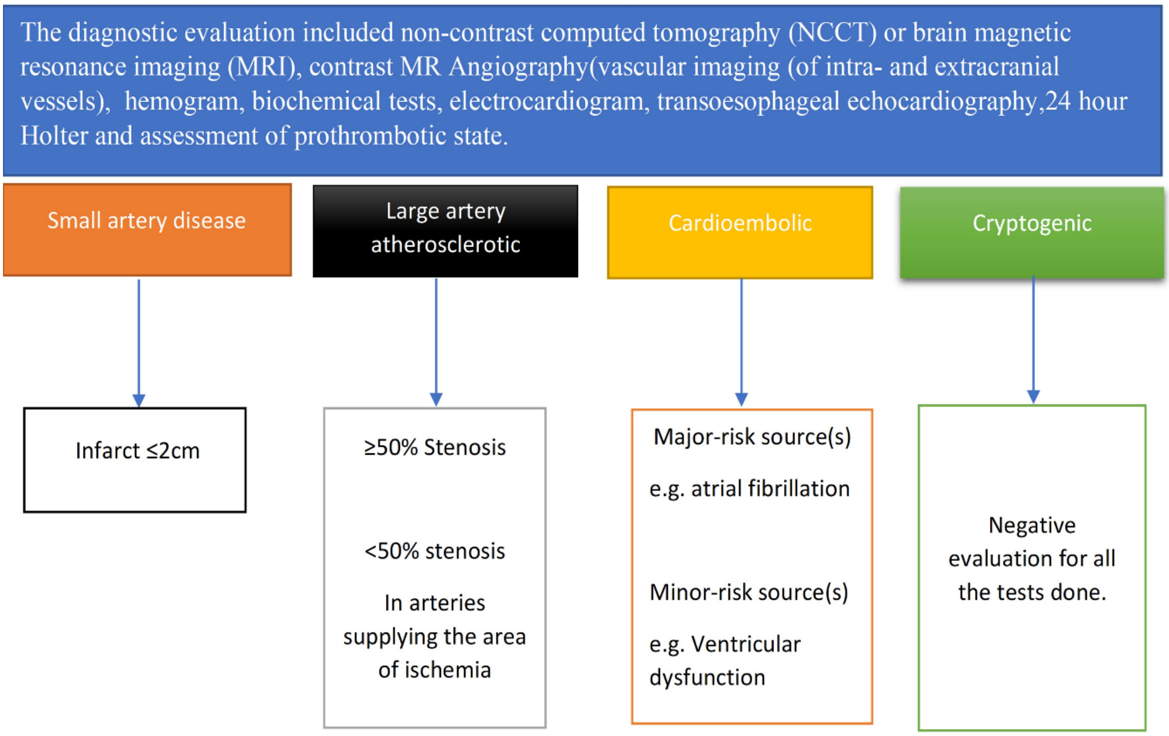

Supplement: Supplementary file 1 — Supplementary Information. [file 41598_2023_32143_MOESM1_ESM.pdf]
